# Case Report: Concurrent transcatheter aortic and mitral valve replacement for symptomatic concomitant aortic and mitral valve stenoses

**DOI:** 10.3389/fcvm.2024.1487061

**Published:** 2024-12-09

**Authors:** Benjamin Mothibe Bussmann, Sam Dawkins, James Newton, Thomas Cahill

**Affiliations:** ^1^ Oxford Heart Centre, Oxford University Hospitals NHS Trust, Oxford, United Kingdom; ^2^ Burdon Sanderson Cardiac Science Centre, Department of Physiology, Anatomy and Genetics, University of Oxford, Oxford, United Kingdom

**Keywords:** TMVR in MAC, TAVI, multi-valve disease, guidelines and recommendations, heart valve team, mitral stenosis (MS)

## Abstract

In patients undergoing transcatheter aortic valve implantation (TAVI), multi-valve disease is common and associated with worse outcomes. Despite multiple emerging transcatheter valve treatment options, no guidelines exist for the transcatheter treatment of multi-valve disease. We present a case of a 76-year-old patient with concomitant severe aortic valve stenosis and severe mitral valve stenosis who underwent concurrent TAVI and transcatheter mitral valve replacement. In this case report, we demonstrate the feasibility of concurrent double-valve transcatheter intervention to treat patients with multi-valve disease. We also highlight the role of the heart valve team to guide individual patient treatment strategies in the absence of clinical guidelines and the importance of multi-modality imaging to plan and execute the procedure.

## Graphical Abstract

Peri-procedural transesophageal echocardiograms showing **(a)** calcified and restricted aortic and mitral valve leaflets before the procedure and **(b)** well-seated Sapien valves in the aortic and mitral positions after the procedure. **(c)** Fluoroscopic image demonstrating the deployed septal occluder device (#) and Sapien valves in the aortic (*) and mitral (@) positions.

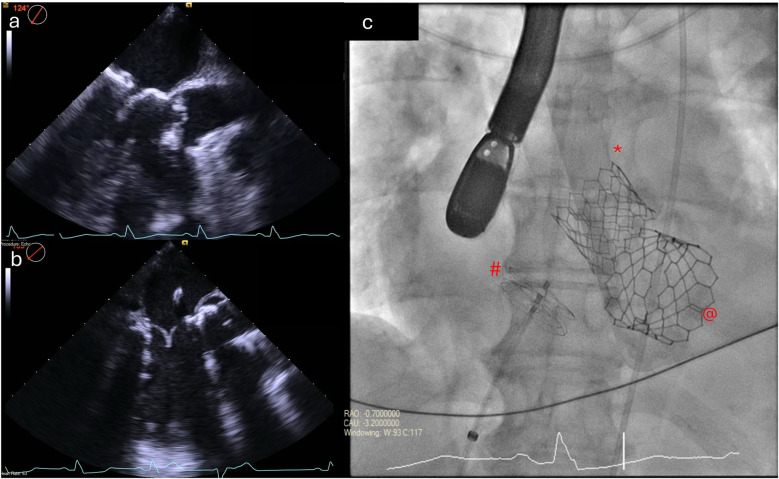

## Introduction

Transcatheter aortic valve implantation (TAVI) has become the dominant mode of treatment for patients with symptomatic severe aortic stenosis and carries an IA indication for patients aged 75 years or over, irrespective of surgical risk (1). Approximately half of patients with valvular heart disease have multi-valve involvement (2), and multi-valve disease (MVD) is common in patients undergoing TAVI, where it is associated with worse outcomes (3). There are ever-expanding treatment options available to treat bystander valve disease. Mitral transcatheter edge-to-edge repair (TEER) for mitral regurgitation has been shown to reduce symptoms and improve outcomes in selected patients (4) and has recently been shown to be non-inferior to valve surgery (5). Similarly, tricuspid TEER reduces tricuspid regurgitation, leading to improved quality of life (6). Transcatheter valve implantation is also possible in the mitral and tricuspid positions, although these procedures are less developed than TAVI (7, 8). With the ever-increasing number of TAVI procedures and transcatheter options to treat bystander disease, transcatheter treatment of patients with MVD will become a common challenge (9). However, while guidelines advocate for concurrent surgical repair of all significant valve lesions (1, 10), no such guidelines exist to guide transcatheter treatment of MVD.

## Case presentation

A 76-year-old woman was referred by her primary care practitioner with exertional breathlessness and worsening lethargy [New York Heart Association (NYHA) stage III]. She denied any anginal symptoms and there was no associated orthopnea or paroxysmal nocturnal dyspnea. Physical examination was notable for a harsh ejection systolic murmur. Her co-morbidities were essential hypertension and chronic obstructive pulmonary disease (COPD).

**Figure 1:**
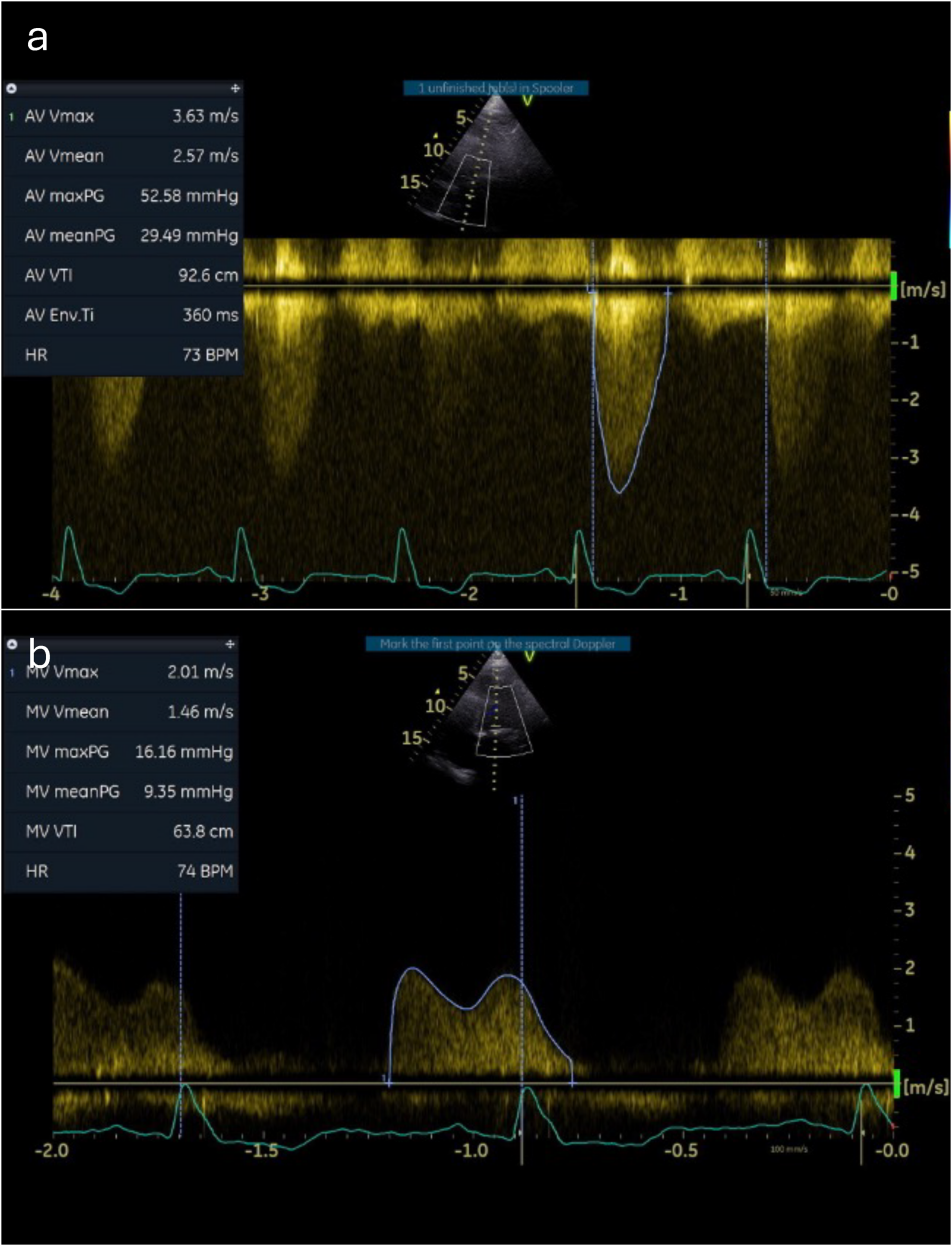
Baseline Doppler tracings across the aortic and mitral valves. Continuous-wave Doppler measurements through the aortic **(a)** and mitral **(b)** valves demonstrate severe stenosis.

**Figure 2:**
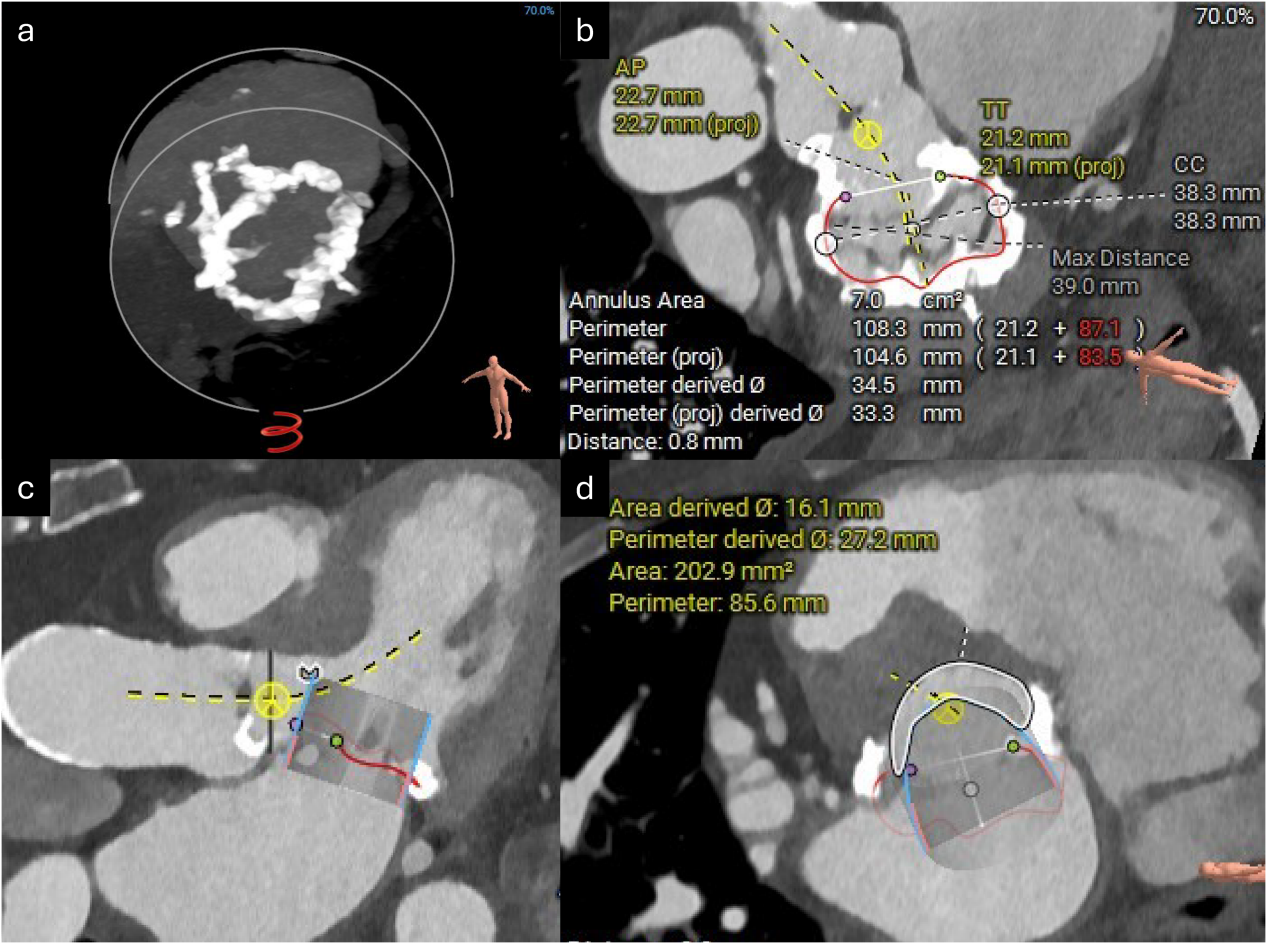
CT reconstructions of the mitral valve annulus. **(a)** 3D reconstruction showing concentric mitral annular calcification. **(b)** Measurements of the mitral annular area. **(c)** Simulated 29 mm Sapien valve in the mitral valve annulus. **(d)** Estimated neo-LVOT area with the simulated Sapien valve *in situ*.

A 12-lead echocardiogram (ECG) demonstrated sinus rhythm with left bundle branch block. Transthoracic echocardiography showed preserved left ventricular systolic function. The aortic valve was tricuspid in morphology with severe low-flow low-gradient aortic stenosis (peak gradient of 52 mmHg, mean gradient of 29 mmHg, aortic valve area of 0.53 cm^2^, and stroke volume index of 17 ml/m^2^). There was severe mitral annular calcification associated with at least moderate mitral stenosis (MS) (mean gradient 9.4 mmHg) and mild mitral regurgitation (MR) (Figure 1, Supplementary Video S1). There was also moderate tricuspid regurgitation (TR) with evidence of elevated pulmonary artery pressure (peak TR gradient of 60 mmHg). Cardiac CT showed no evidence of obstructive coronary artery disease but revealed very heavy calcification of the ascending aorta (so-called “porcelain” aorta) and concentric calcification of the mitral valve annulus (Figure 2).

Her case was brought to the heart valve team for discussion. Based on the patient’s porcelain aorta, established COPD, and pulmonary hypertension, her risk for cardiac surgery was deemed to be prohibitive by the heart valve team (EuroSCORE II of 11%). In the context of the low-flow state caused by the tandem aortic and mitral valve stenoses, it was felt that a mean mitral valve gradient of 9.4 mmHg was consistent with severe MS. There was thus consensus that both the aortic and mitral valves required intervention on symptomatic and prognostic grounds. She was thus put forward for TAVI and transcatheter mitral valve replacement (TMVR) through valve-in-mitral annular calcification (ViMAC).

**Figure 3:**
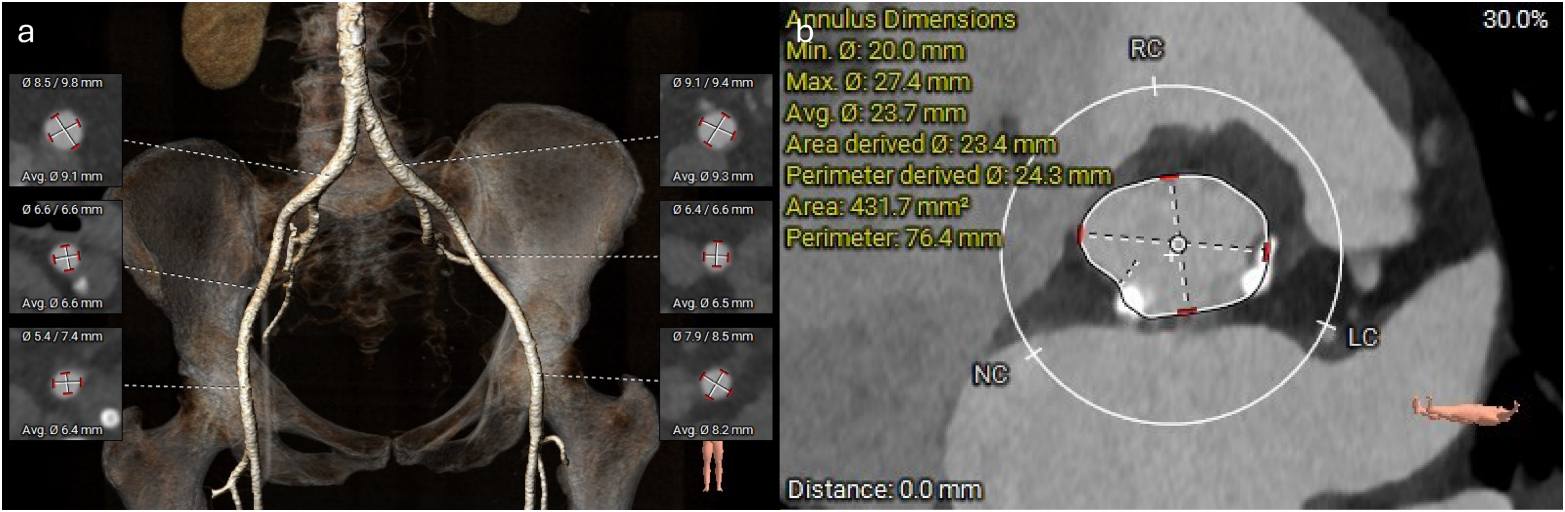
CT reconstructions of the femoral vessels and aortic annulus. **(a)** 3D reconstruction of the femoral arterial access. **(b)** Measurements of the aortic annulus.

A TAVI protocol CT scan demonstrated good-caliber iliofemoral vessels for a transfemoral approach. The aortic annulus measured 432 mm^2^ with adequate coronary heights (Figure 3). A 26 mm Sapien 3 Ultra valve (Edwards Lifesciences) was selected for transfemoral TAVI. Next, the mitral annulus and left ventricular outflow tract (LVOT) were assessed for feasibility of mitral ViMAC. First, the circumferential distribution and degree of the annular calcification were deemed sufficient for valve anchoring with a low risk of valve embolization. Next, the risk of LVOT obstruction post-valve implantation was deemed to be low: there was no significant calcification of the sub-valvular apparatus or the anterior mitral valve leaflet, there was no hypertrophy of the basal septum, and the predicted minimum neo-LVOT area after deployment of a 29 mm Sapien 3 valve was 203 mm^2^ (Figure 2). Thus, no routine adjunctive procedures to modify the LVOT area (e.g., alcohol septal ablation or electrosurgical laceration of the anterior mitral valve leaflet) were planned. The mitral annular area was measured at 700 mm^2^ so a 29 mm Sapien 3 valve (Edwards Lifesciences) was selected for TMVR via a transseptal approach. Since the mitral stenosis was unlikely to improve after TAVI, delaying mitral valve intervention was deemed unnecessary, and thus TAVI with mitral ViMAC was planned to be performed in a single procedure. Given the favorable anatomy on CT, it was felt that this could be achieved without additional procedural risk and would improve the patient’s experience and prevent recurrent hospital admissions between procedures.

Concurrent TAVI and TMVR (ViMAC) were performed under general anesthesia with transesophageal echocardiographic (TEE) guidance. Vascular access was gained using a micropuncture access set (Cook Medical) under ultrasound guidance. Initially, 6 F access sheaths were inserted into the right radial artery, right common femoral vein (CFV), and left CFV and a 9 F sheath was inserted into the right common femoral artery (CFA). A temporary pacing wire was inserted from the left CFV. Heparin was given with an activated coagulation time target of 300 s.

TAVI: The right CFA was used as the primary arterial access and was upgraded by inserting a 14 F eSheath (Edwards Lifesciences) over a Lunderquist wire (Cook Medical). Secondary arterial access via the right radial artery was used to position a pigtail catheter in the aortic root. The aortic valve was crossed using a straight wire in an AL-1 catheter. The straight wire was then exchanged for a small Safari pre-shaped wire (Boston Scientific), which was advanced into the left ventricular cavity. The 26 mm Sapien 3 Ultra valve was introduced over the Safari wire and positioned in the aortic annulus using fluoroscopy. The final valve position was confirmed with an aortogram and the valve was then deployed under rapid ventricular pacing. A repeat aortogram after the valve deployment confirmed good valve position, patent coronary arteries, and only trace aortic regurgitation, and there was no change in the PR interval or QRS morphology on ECG.

**Figure 4:**
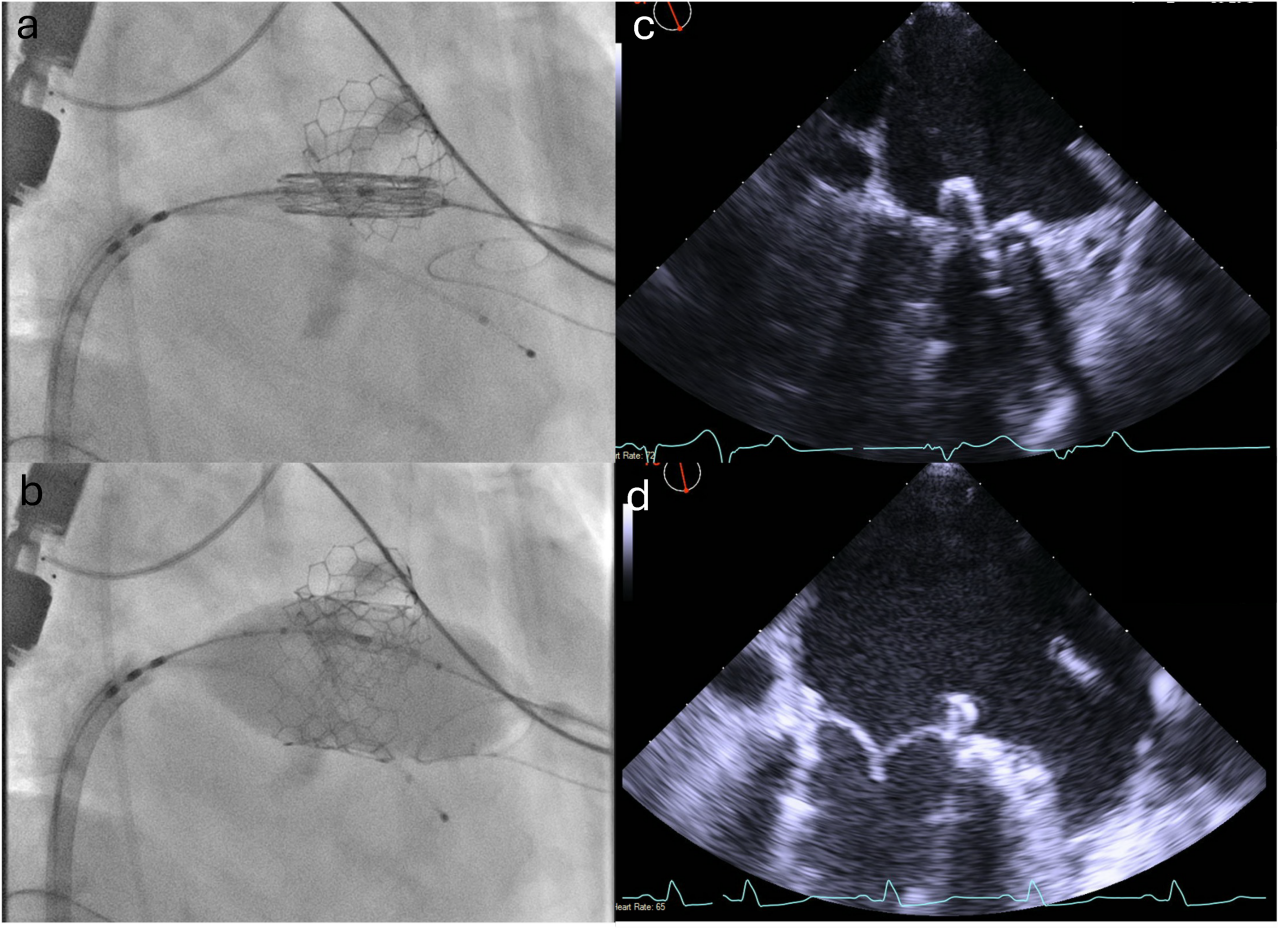
Positioning and deployment of the Sapien valve in the mitral position. Fluoroscopic **(a,b)** and TEE **(c,d)** images demonstrating positioning (top panels) and deployment (bottom panels) of the Sapien valve in the mitral position.

TMVR (ViMAC): The right CFV was used as the primary venous access. The 6F sheath was exchanged for a 16 F eSheath which was inserted over a Supracore wire (Abbott Cardiovascular). The TEE-guided transseptal puncture was performed using the VersaCross transseptal system (Baylis Medical) in a mid-posterior position on the fossa ovalis to allow adequate height above the mitral annulus to maneuver the valve into position. An Agilis catheter (Abbott Cardiovascular) was then advanced over the VersaCross wire into the left atrium and used to steer down toward the mitral annulus. A 6 F pigtail catheter was advanced across the mitral valve into the left ventricle and used to introduce a small Safari pre-shaped wire. The interatrial septum was dilated with a 14 mm × 60 mm EverCross balloon (Medtronic) to facilitate transseptal delivery of the Sapien valve. The 29 mm Sapien 3 valve was crimped and mounted on the delivery system in the inverted position, and then positioned in the mitral annulus under TEE and fluoroscopic guidance (Figure 4), aiming for approximately 40% atrial and 60% ventricular positioning. The valve was deployed slowly to allow coaxialization within the annulus under rapid pacing. After the valve deployment, TEE confirmed a good valve position with no paravalvular leak (Supplementary Videos S2 and S3). The patient’s post-deployment mean mitral valve gradient was 4 mmHg on TEE and there was only a mild gradient across the LVOT (peak gradient of 25 mmHg). After the removal of the transseptal delivery system, there was a persistent bidirectional shunt across the atrial septum, so the iatrogenic atrial septal defect was sealed with a 25 mm Cardioform septal occluder (Gore Medical). At the end of the procedure, the right CFA access site was closed using two Perclose ProGlide sutures (Abbott Cardiovascular), and the right CFV was closed with one Perclose ProGlide suture, with good hemostasis.

The patient was successfully extubated in the catheter laboratory and had an uneventful recovery in the coronary care unit. She was discharged 48 h later on apixaban 5 mg b.i.d.

### Follow-up

At the 3-month follow-up, there was marked symptomatic improvement with increased exercise capacity (now NYHA I) and improved energy levels. A transthoracic echocardiogram confirmed well-functioning prosthetic valves in the aortic (mean gradient 7 mmHg, peak gradient 13 mmHg) and mitral positions (mean gradient 7 mmHg) with only trace MR.

## Discussion

MVD is common in patients undergoing TAVI, with concomitant mitral valve disease being the most common. Up to 36% of patients undergoing TAVI have moderate to severe MR (3) and a further 18% have MS (11). Both MR and MS are associated with increased mortality following TAVI (11, 12). While TAVI can lead to a reduction in MR severity in some cases, up to 50% of MR cases do not improve or even worsen (13).

Numerous transcatheter options are now available to treat MV disease (14, 15), leading many to advocate for a combined transcatheter aortic and mitral valve intervention (3, 16). Observational data suggest this approach is safe and may improve outcomes (17). However, the optimal strategy for combining mitral and aortic valve interventions (staged vs. concurrent procedures) remains unclear (18). In the absence of guidelines, consulting with a multidisciplinary heart valve team to guide treatment on an individual patient basis is crucial. Based on limited observational data, the treatment strategy is currently guided by the etiology of the mitral valve disease and factors that may predict the improvement in bystander valve disease following TAVI (16, 19). Because MS due to mitral annular calcification was unlikely to improve following TAVI in this case, we opted to perform both TAVI and TMVR as concurrent procedures.

Our case illustrates the feasibility of a concurrent transcatheter double-valve intervention in highly selected patients with MVD. This was achieved without the need for an intensive care bed and with only a short hospital stay. We highlight the role of the heart valve team in guiding the treatment strategy and the importance of multi-modality imaging during the planning and execution of the procedure.

### Learning objectives

1. To understand the prognostic importance of MVD in patients undergoing TAVI
2. To appreciate the possibility of concurrent transcatheter double-valve intervention for MVD in patients with prohibitive surgical risk
3. To appreciate the importance of the heart valve team and multi-modality imaging in planning transcatheter treatment for patients with MVD.

## Data Availability

The original contributions presented in the study are included in the article/Supplementary Material, further inquiries can be directed to the corresponding author.

## Supplementary material

Supplementary Video S1 Baseline transthoracic echocardiogram. Parasternal long-axis view demonstrating calcification and severe restriction of the aortic and mitral valve leaflets.

Supplementary Video S2 Post-procedural transesophageal echocardiogram. X-plane images demonstrating well-seated Sapien valves in the aortic and mitral positions.

Supplementary Video S3 3D transesophageal echocardiogram. 3D TEE reconstruction showing a well-functioning Sapien valve in the mitral annulus.
